# Non-classical nucleation in vapor–liquid–solid growth of monolayer WS_2_ revealed by in-situ monitoring chemical vapor deposition

**DOI:** 10.1038/s41598-021-01666-9

**Published:** 2021-11-15

**Authors:** Xiaoming Qiang, Yuta Iwamoto, Aoi Watanabe, Tomoya Kameyama, Xing He, Toshiro Kaneko, Yasushi Shibuta, Toshiaki Kato

**Affiliations:** 1 Graduate School of Engineering, Tohoku University, Sendai, 980-8579 Japan; 2 Department of Materials Engineering, The University of Tokyo, 7-3-1, Hongo, Bunkyo-ku, Tokyo 113-8656 Japan

**Keywords:** Synthesis of graphene, Synthesis of graphene, Two-dimensional materials, Synthesis and processing

## Abstract

The very early nucleation stage of a transition metal dichalcogenide (TMD) was directly observed with in-situ monitoring of chemical vapor deposition and automated image analysis. Unique nucleation dynamics, such as very large critical nuclei and slow to rapid growth transitions, were observed during the vapor–liquid–solid (VLS) growth of monolayer tungsten disulfide (WS_2_). This can be explained by two-step nucleation, also known as non-classical nucleation, in which metastable clusters are formed through the aggregation of droplets. Subsequently, nucleation of solid WS_2_ takes place inside the metastable cluster. Furthermore, the detailed nucleation dynamics was systematically investigated from a thermodynamic point of view, revealing that the incubation time of metastable cluster formation follows the traditional time–temperature transformation diagram. Quantitative phase field simulation, combined with Bayesian inference, was conducted to extract quantitative information on the growth dynamics and crystal anisotropy from in-situ images. A clear transition in growth dynamics and crystal anisotropy between the slow and rapid growth phases was quantitatively verified. This observation supports the existence of two-step nucleation in the VLS growth of WS_2_. Such detailed understanding of TMD nucleation dynamics can be useful for achieving perfect structure control of TMDs.

Transition metal dichalcogenides (TMDs) are among the most well-known layered materials^1–6^. They have various features that are desirable in semiconductors^1,2^—for example, stable neutral and charged excitons^3,4^, valley polarization capability^5^, and superconductivity^6^. From a historical perspective, establishing ultra-high yield synthesis techniques, such as super growth in carbon nanotubes^7^ and large-scale growth of monolayer graphene on Cu foil^8^, have accelerated the study of nanomaterials. Recently, similar progress has been made for TMDs through salt-assisted growth^9^, which enables the growth of ultra-large (mm)-scale single-crystal monolayer TMDs with high reproducibility. Efforts to understand the growth mechanism of this salt-assisted growth have concluded that the vaporization of metal oxide source powder (MO_x_) can be enhanced by salt assistance through the lowering the melting and boiling points of MO_x_ (Fig. 1a)^9^. Supersaturation of MO_x_ in the vapor phase promotes the creation of liquid-phase precursors, which promote vapor–liquid–solid (VLS) growth over conventional vapor–solid (VS) growth (Fig. 1a)^10^. Alkali metal salts can act as catalysts to decrease the energy barrier and increase the surface reaction rate^11^. By introducing lattice distortion and reducing the activation energy on the specific surface of non-layered materials, growth perpendicular to the surface can be suppressed to promote two-dimensional (2D) growth^11^. The growth rate of VLS-grown TMD domains is at least two orders of magnitude higher than that of VS-grown TMD domains^12^. Despite this progress, the critical dynamics of the nucleation phase has not yet been elucidated for salt-assisted growth; achieving this is crucial for both fundamental and industrial applications. Recently, we established in-situ monitoring of chemical vapor deposition (CVD) in TMD synthesis, revealing the ultra-long diffusion of liquid phase precursors and the existence of a precursor puddle^13^. However, the critical question of how these phenomena contribute to the nucleation of TMDs remains.

**Figure 1:**
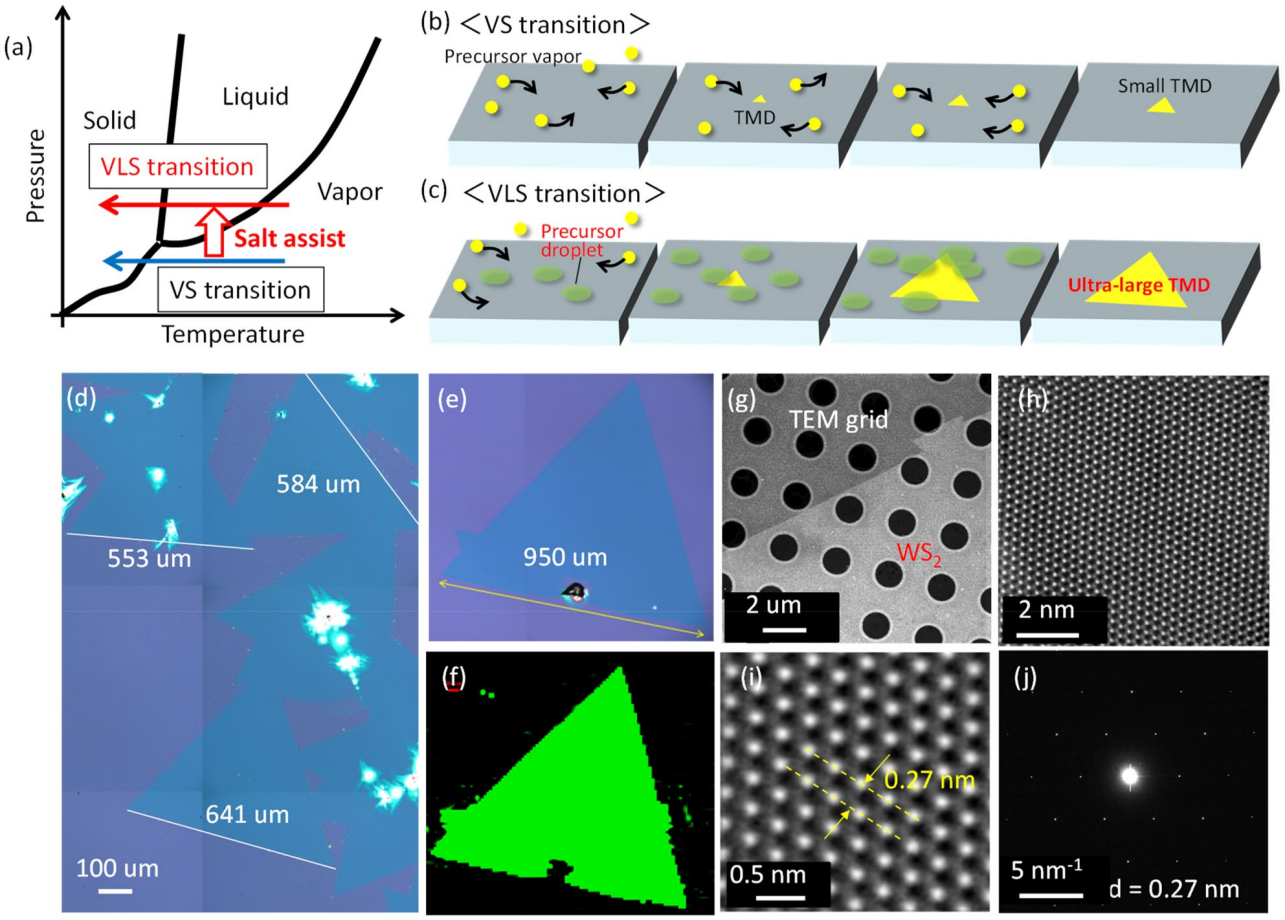
Comparison between VS and VLS transition. (**a**) Schematic of phase transition for non-salt assisted (VS transition) and salt-assisted (VLS transition) growth. (**b**, **c**) Difference of TMD growth between (**b**) VS transition and (**c**) VLS transition. (**d**–**h**) Typical (**d**) low and (**e**) high magnification optical microscope image, (**f**) photoluminescence (PL) mapping image, (**g**) low magnification TEM image, (**h**) low and (**i**) magnification of STEM images, (**j**) electron diffraction pattern image of monolayer WS_2_ grown with our salt-assisted CVD.

In this study, we realized the direct visualization of the phase transition from liquid precursors to solid TMD through in-situ monitoring CVD and automated image analysis. Critical nuclei, the initial transition from embryo to nuclei at the very early crystal growth stage, were directly observed. The experimental results can be explained by two-step nucleation, also known as non-classical nucleation^14,15^. The nucleation dynamics of TMD were systematically investigated from a thermodynamic point of view. Furthermore, a combination of quantitative phase field simulations (Q-PFS) and in-situ imaging using data assimilation techniques was carried out. This enabled us to quantitatively discuss the time evolution of growth dynamics and crystal anisotropy, and our observations support the occurrence of non-classical nucleation. These findings can contribute to improving the quality of TMD crystals, which would be useful for future industrial applications.

Typical monolayer and single-crystal tungsten disulfide (WS_2_) grown by salt-assisted CVD are shown in Fig. 1d–j. We chose WS_2_ as a typical example to explain the growth model. Since type of starting materials (metal oxide (MO_x_), salt (Sal), chalcogen(Cha)) and basic reaction (MO_x_ + Sal + Cha → TMD) should be quite similar, the proposed WS_2_ growth model shown here can be applicable for other type of TMD such as MoS_2_, MoSe_2_, WSe_2_, and so on. Further detailed experimental set up is shown in Fig. S1. An almost millimeter-scale large single-crystal WS_2_ can be grown in our system (Fig. 1d–f). The single crystalline structure of the triangular domain was confirmed by atomic-resolution Z-contrast scanning transmission electron microscopy (STEM). The periodic W atom can be clearly observed with a 0.27 nm space (Fig. 1h,i), which is consistent with the lattice constant of WS_2_. The electron diffraction patterns in Fig. 1j show one set of hexagonal symmetrical patterns, indicating that the relatively high-quality single crystal of WS_2_ can be grown by salt-assisted CVD. These results are consistent with those of a previous report on the salt-assisted growth of TMD^9^, demonstrating that the effects of salt assistance can be accurately investigated with our CVD system.

In-situ monitoring of CVD is a powerful tool that enables optical imaging of the substrate during growth in real time^13,16,17^. In our in-situ monitoring CVD system, 300 pictures (1/s) were captured for every single CVD run. The time evolution of single crystal growth of monolayer TMD can be observed clearly with this in-situ monitoring CVD (Movie 1). To efficiently collect information on crystal growth, we established an automated image analysis system (see the “Methods” section for more details), in which a specific region where the color index is the same as a predefined value can be automatically extracted from all optical images. By changing the predefined threshold values of the hue, saturation, value (HSV) color index, the monolayer and multilayer regions can be independently extracted from the original images (Fig. S2). We can also obtain the area plot of monolayer and multilayer as a function of growth time to extract important physical parameters for crystal growth, such as incubation time ($\Delta t$) and growth speed ($v_{g}$) (Fig. S3). Using this automated image analysis, detailed investigations were carried out for the initial nucleation phase for WS_2_ growth (Fig. 2a,b). By extracting and highlighting the edge of the monolayer color region, it was found that many particles existed on the substrate (Fig. 2b), whereas no clear structures could be identified with the naked eye in the original images (Fig. 2a). Note that the size of the particles is much larger than the single pixel size of our CCD detector, denoting that the identified particles are not pixel noise but physical structures (Fig. S4). These particles appear to move on the surface, and the crystal size increases through the collision of the particles (Movie 2), indicating that the particles should be liquid-phase precursors, which is consistent with the conclusions of our previous study^13^. When precursors are oversupplied, the moving liquid edge can also be directly observed in real-time images (Fig. S5). These results support the conjecture that the salt-assisted growth of TMD should be conducted not by conventional VS growth (Fig. 1b) but by VLS growth (Fig. 1c). When we plotted the area of the monolayer as a function of growth time, we observed that the area of the monolayer increased after 150 s (Fig. 2c). However, crystal-like structures can be identified from optical images even before 150 s (140–150 s) (Fig. 2b(ii–iv)). When we carefully focused on this period (Fig. 2f), we observed that the cluster size gradually increased with repeated collisions of additional particles after 140 s (Fig. 2f(iii–v)), showing that nucleation occurred around this time. By using classical nucleation theory, the difference in the total Gibbs free energy ($\Delta G$) during the nucleation of a single-crystal TMD can be expressed by the following equations^18,19^:

1 $$ \Delta G = \frac{\sqrt 3 }{4}L^{2} \Delta G_{V} + 3L\sigma , $$

2 $$ \Delta G_{V} = G_{S} - G_{X} , $$

where $$L$$, $$\sigma$$, $$G_{S}$$, $$G_{X}$$, and $$\Delta G_{V}$$ denote the crystal size (length of triangle edge) of the TMD, surface energy per unit length, free energy of solid and $$X$$ (liquid or vapor) state, and its difference respectively. The first and second terms in Eq. (1) represent the gain of free energy through the liquid-to-solid phase transition (volume energy) and the penalty of surface energy by crystal formation (interfacial energy), respectively (Fig. 2d). Only when the differential of $$\Delta G$$ ($$d\Delta G / dL$$) is negative can crystal size increase, *that is,* crystals can be grown (Fig. 2e). The threshold crystal size is known as the critical nuclei size, $$r^{*} = \left( {{\raise0.7ex\hbox{${ - 2\sqrt 3 \sigma }$} \!\mathord{\left/ {\vphantom {{ - 2\sqrt 3 \sigma } {\Delta G_{V} }}}\right.\kern-\nulldelimiterspace} \!\lower0.7ex\hbox{${\Delta G_{V} }$}}} \right)$$^18,19^. Since the cluster size starts increasing from 140 s onwards, the cluster size at 140 s should correspond to $$r^{*}$$, which was found to reach up to 38.7 µm (Fig. 2f(iii)). This is much larger than the calculated value for W_15 ± 1_Se_28 ± 3_ ($$r^{*}$$ = 1.63 ± 0.21 nm)^20^ or traditional bulk 3D crystal (< few tens nm)^21,22^; thus, it is difficult to explain this with classical nucleation theory only. It has also been reported that $$r^{*} $$ depends on the size of the precursor. If the precursor size is not usually a single atomic level but much larger (~ µm) order like in particle cluster nucleation, the $$r^{*} $$ can reach up to several tens of µm^23^. However, even in this situation, the area of the crystal should continuously increase after reaching the critical threshold ($$L = r^{*}$$) under a constant precursor supply, which is not consistent with our experimental results (Fig. 2c).

**Figure 2:**
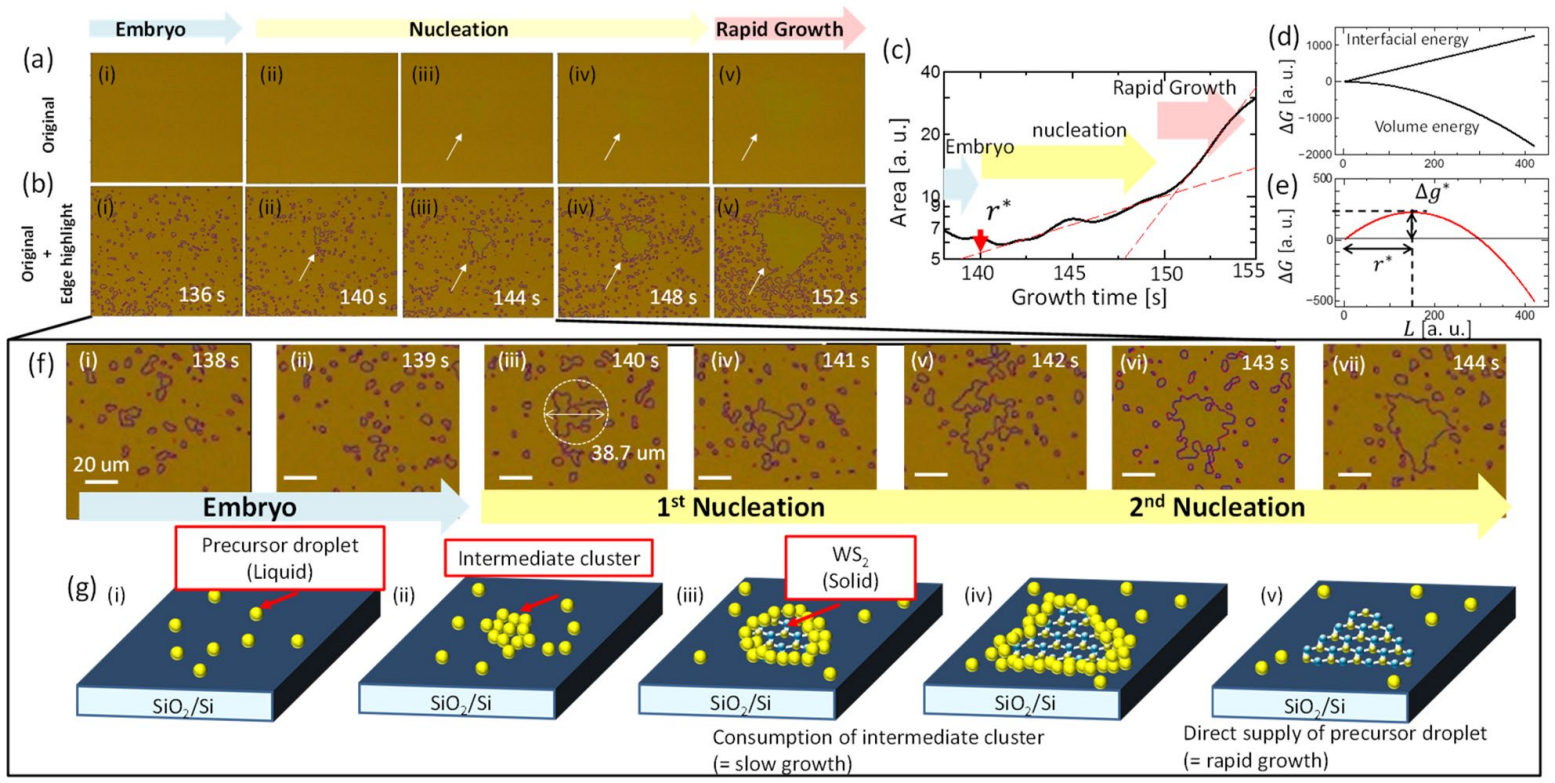
In-situ monitoring CVD. (**a**, **b**) Typical (**a**) original and (**b**) analyzed images of WS_2_ growth obtained by in-situ monitoring CVD at different growth time (i: 136 s, ii: 140 s, iii: 144 s, iv: 148 s, v: 152 s). (**c**) Plot of monolayer area as a function of growth time. Two dashed lines show the different growth speed during the 1st–2nd nucleation and rapid growth period. (**d**, **e**) Calculated curves of (**d**) volume energy gain, interfacial energy loss, and (**e**) $\Delta G$ as a function of $L$. (**f**) Time profile of analysed images between (i) 138 s and (vii) 144 s. (**g**) Schematic illustration of two-step nucleation of WS_2_ (i: precursor supply, ii: 1st nucleation of intermediate cluster, iii: 2nd nucleation of WS_2_ within intermediate cluster, iv: WS_2_ growth within intermediate cluster, v: WS_2_ growth during rapid growth).

Thus, we focus on non-classical nucleation to explain this micrometer order of $$r^{*} $$. It has been reported that a type of non-classical nucleation, called two-step nucleation, can be applied to explain complex materials such as proteins, colloids, minerals, and polymeric solutions^14,15,24,25^. In classical nucleation (one-step nucleation), the precursors in the liquid are directly transferred to the crystal. In contrast, in two-step nucleation, the precursor in the liquid tends to form an intermediate state, which is then transferred to the solid crystal. In this case, the crystal area slowly increases at the initial nucleation stage because the phase transition occurs inside the intermediate clusters, and the relatively low mobility of such intermediate clusters limits the speed of crystal growth to a low value^24,25^. Once most of the intermediate clusters are consumed for the crystal growth, the growth point (edge) of the crystal becomes open for the movable precursor droplet existing in the bulk liquid, thus increasing the speed of crystal growth^24,25^. This explanation for the time evolution of the two-step nucleation matches well with our experimental results. Nucleation occurred at 140 s, and then slowly increased between 140 and 150 s (Fig. 2g(ii)–(iv)). After 150 s, the crystal size rapidly increased (Fig. 2g(v)). Thus, it is reasonable to assume that the relatively large $$r^{*} $$ observed in this study can be attributed to the nuclei of the intermediate cluster of WS_2_. The real nucleation of WS_2_ may occur after this intermediate nucleation (between 140 and 150 s from a much smaller critical nuclei size as shown in Fig. 2g(iii)), that is, two-step nucleation occurring during the VLS growth of TMD. The precursor puddle observed in our previous study should also be part of this two-step nucleation^13^. It should also be mentioned that WS_2_, being much smaller crystal size than 38.7 µm (several hundred nanometer), can often be observed without salt assistance (Fig. S6). This indicates that the $$r^{*}$$ obtained without salt assistance should be much smaller than that obtained with salt assistance. These results indicate that non-classical nucleation is a unique feature of VLS growth in salt-assisted TMD synthesis.

To further understand the non-classical nucleation in the VLS growth of TMDs, systematic investigations were carried out. If we assume that this liquid–solid phase transition in TMD is similar to that in other bulk crystals, this reaction can be expressed by the solidification reaction (Fig. 3a,b), where the driving force ($F$) of TMD growth depends on the chemical potential difference ($\Delta \mu$) between liquid ($\Delta \mu_{L}$) and solid ($\Delta \mu_{S}$). This, in turn, is influenced by the temperature difference ($\Delta T$) between the undercooled liquid and the melting point of the solid crystal ($T_{m}$) (Fig. 3b)^18,19^. It can be assumed that the liquid temperature is the same as that of the substrate whereas the solid (TMD) temperature is always higher than that of the liquid because of the heat of crystallization. We also attempted to investigate the effects of $\Delta T$ by introducing a spot heater to control the temperature of the liquid precursor on the substrate, independent of other conditions such as temperature of the main furnace (vapor temperature), gas flow, and substrate position (Fig. S1).

**Figure 3:**
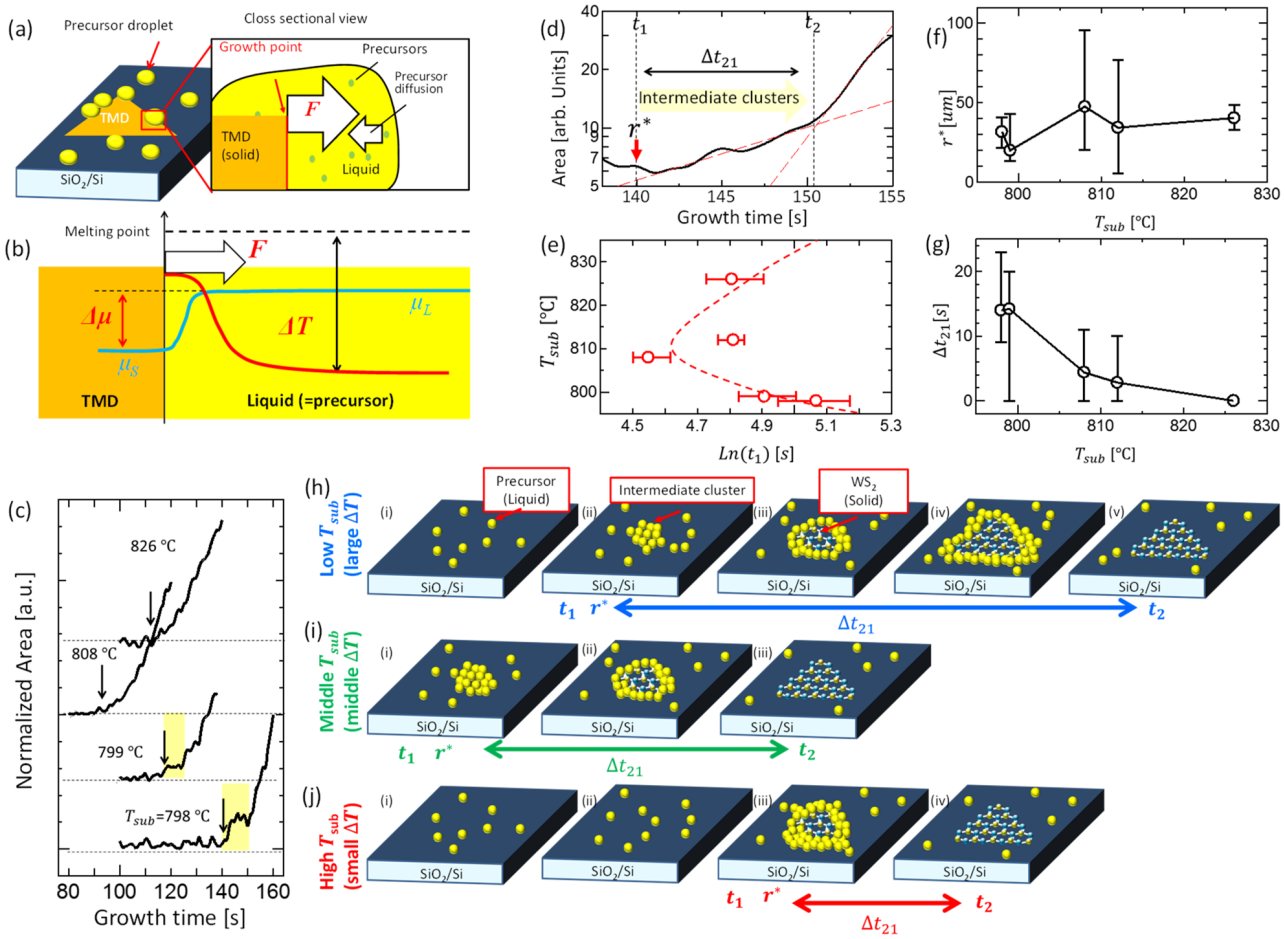
Thermodynamic approach for WS_2_ nucleation. (**a**,**b**) Schematic of (**a**) WS_2_ growth through the coagulation reaction and (**b**) enlarged interface between TMD and liquid precursor. (**c**) Time evolution of monolayer area of WS_2_ grown under the different $$T_{sub}$$. Arrow shows the $$t_{1} $$ for each growth. (**d**) Redefinition of parameters within the curve of area versus growth time at initial growth stage. (**e**) Plot of $$T_{sub} - {\text{Ln}}\left( {t_{1} } \right)$$. Dashed line is guid for eye. (**f**,**g**) Plot of (**g**) r* and (**h**) $$\Delta t_{21}$$ as a function of $$T_{sub}$$. (**h**–**j**) Schematic illustration of two-step nucleation of WS_2_ under the different $$T_{sub}$$ (**i**: Low $$T_{sub}$$, **j**: Middle $$T_{sub}$$, **k**: high $$T_{sub}$$).

We carried out similar in-situ monitoring and auto image analysis to measure the $r^{*}$ under different substrate temperatures ($T_{sub}$), which were controlled by the spot heater output (Fig. S1). Here, we redefine the nucleation phase (Fig. 3d). The time of nucleation of the intermediate cluster and the starting time of rapid growth are defined as *t*_1_ and *t*_2_ respectively. The time difference between *t*_1_ and *t*_2_ is shown as $\Delta t_{21}$, which corresponds to the time required to consume intermediate clusters for WS_2_ crystal growth. The time profile of the monolayer area grown under different $T_{sub}$ values is shown in Fig. 3c. It was found that *t*_1_ and *t*_2_ are strongly influenced by $T_{sub}$. The nucleation speed ($v_{n}$) should be inversely proportional to $\Delta t \left( {v_{n} \propto 1 / \Delta t} \right)$, which can be expressed by the product of the precursor diffusion and driving force terms for the growth^18,19^.

3 $$ v_{n} \propto exp\left( { - \frac{{\Delta G_{m} }}{kT}} \right)exp\left( { - \frac{{\Delta g^{*} }}{kT}} \right), $$

where $k, \Delta G_{m} ,T,\;and\;\Delta g^{*}$ denote the Boltzmann constant, activation energy for one atom movement, temperature, and energy barrier for nucleation, respectively. The diffusion term increases exponentially with $T$, whereas the driving force term decays exponentially with $T$ since $\Delta g^{*}$ is proportional to the inverse of the square of the undercooling temperature. From Eq. (3), a C-shaped curve ${\text{ln}}\left( {\Delta t} \right)$ versus $T$ plot can be drawn, which is the temperature–time transformation (TTT) diagram^26^. A ${\text{ln}}\left( {\Delta t} \right)$ versus $T$ plot of our data shows that both *t*_1_ and *t*_2_ matches well with those of the TTT diagram (Fig. 3e and Fig. S7). This indicates that at least the nucleation dynamics of the intermediate clusters can be explained by the traditional TTT diagram. The balance of diffusion of the precursor on the substrate and the driving force for the growth is thus important.

The critical nuclei size of the intermediate cluster $$r^{*}$$ gradually increases with $$T_{sub}$$ (Fig. 3f), which can also be explained well by the traditional theory. As discussed above, $$r^{*} = \left( {{\raise0.7ex\hbox{${ - {2}\sqrt {3} \sigma }$} \!\mathord{\left/ {\vphantom {{ - {2}\sqrt {3} \sigma } {\Delta G_{V} }}}\right.\kern-\nulldelimiterspace} \!\lower0.7ex\hbox{${\Delta G_{V} }$}}} \right)$$ and $$\Delta G_{V} = \frac{{L\left( {T_{m} - T_{sub} } \right)}}{{T_{m} }}$$, where $$L$$ is the latent heat of crystallization. Thus, when $$T_{sub}$$ increases $$\left| {\Delta G_{V} } \right|$$ decreases, resulting in an increase of $$r^{*}$$^18,19^, which is consistent with the experimental results (Fig. 3f) and can be explained by schematic models shown in Fig. 3h–j. The overall tendency of *t*_2_ is the same as *t*_1_, which is natural because *t*_2_ >> $$\Delta t_{21}$$ and *t*_2_ is mainly decided by *t*_1_. However, in the case of $$\Delta t_{21}$$, a unique tendency for $$T_{sub} $$ can be observed. The $$\Delta t_{21}$$ clearly decreased with $$T_{sub}$$ (Fig. 3g). This can be explained using the thermal activation model. The diffusion coefficient of the precursor in the intermediate cluster can be increased by $$T_{sub}$$ owing to simple thermal activation, which can accelerate the consumption of intermediate clusters, resulting in a short $$\Delta t_{21}$$ (Fig. 3j). This is also consistent with the hypothesis that slow diffusion of the precursor in the intermediate state governs the relatively slow growth rate in this initial nucleation stage. Note that any WS_2_ crystal could not be grown on the substrate in the case of high temperature growth (over 850 deg C). This should be because the substrate temperature is very close to the melting point of WS_2_.

As we demonstrated, in-situ monitoring growth under the precise control of $T_{sub}$ can provide insightful information about the nucleation dynamics of TMD, such as $r^{*}$, $t_{1}$, $t_{2}$, and $\Delta t_{21}$. By using this time evolution of in-situ images, further steps were implemented to extract physical parameters relating to the TMD growth. This refers to the data assimilation of computer simulation and experimentally obtained in-situ monitoring images based on Bayesian inference (Fig. 4a). For computer simulation, we used quantitative phase-field simulation (Q-PFS)^27^. In Q-PFS, the dynamics of crystal growth are characterized by the time evolution of the distribution of an order parameter (see “Methods” section for a more detailed explanation). Q-PFS requires thermodynamic and interfacial parameters for target materials, which often makes it difficult to apply Q-PFS for a system with unknown parameters. Therefore, we used “data assimilation,” where parameters such as kinetic coefficient ($\beta_{0}$) and kinetic anisotropy $(\varepsilon_{k} )$ can be estimated from experimental data based on Bayesian inference with the ensemble Kalman filter (EnKF) (Fig. 4a) (see “Methods” section and Fig. S8 for a more detailed explanation)^28,29^. Through this data assimilation of Q-PFS and in-situ monitoring images, the experimentally obtained time evolution of optical images for single-crystal WS_2_ growth, such as shape, size, and speed, were well represented (Fig. 4b and Fig. S9). To the best of our knowledge, this is the first time that data assimilation has been applied to nanomaterial synthesis. Note that we use the time period between 143 and 152 s (Fig. 4d), which corresponds to the transition period from nucleation to rapid growth. We can extract the interfacial parameters using data assimilation for the quantitative discussion of growth dynamics. First, to check on the accuracy of this approach, we focused on $\beta_{0}$, which is proportional to the inverse of $v_{g}$. As shown in Fig. 4e, the dynamics of $\beta_{0}$ are consistent with the experimental data of $1 / v_{g}$. This indicates that accurate discussion should be possible with the extracted parameters from the data assimilation of Q-PFM and in-situ monitoring images. Then, we focused on the other parameter, kinetic anisotropy ($\varepsilon_{k}$), which corresponds to that of the TMD crystal. The triangular and circle-like shapes show high and low $\varepsilon_{k}$, respectively (Fig. 4c). Because it is very difficult to qualitatively discuss the anisotropy of TMD from experimental data, using data assimilation to obtain $\varepsilon_{k}$ is useful for understanding the growth dynamics. When we plot the time evolution of $\varepsilon_{k}$, it is seen that $\varepsilon_{k}$ has a relatively low value during the nucleation phase (144–148 s) (Fig. 4f). Then, the anisotropy gradually increases at almost the same time as the start of the rapid growth (Fig. 4f). This two-step change of $\varepsilon_{k}$, revealed by the data assimilation approach, may also be correlated with the two-step nucleation, as shown in Figs. 2 and 3.

**Figure 4:**
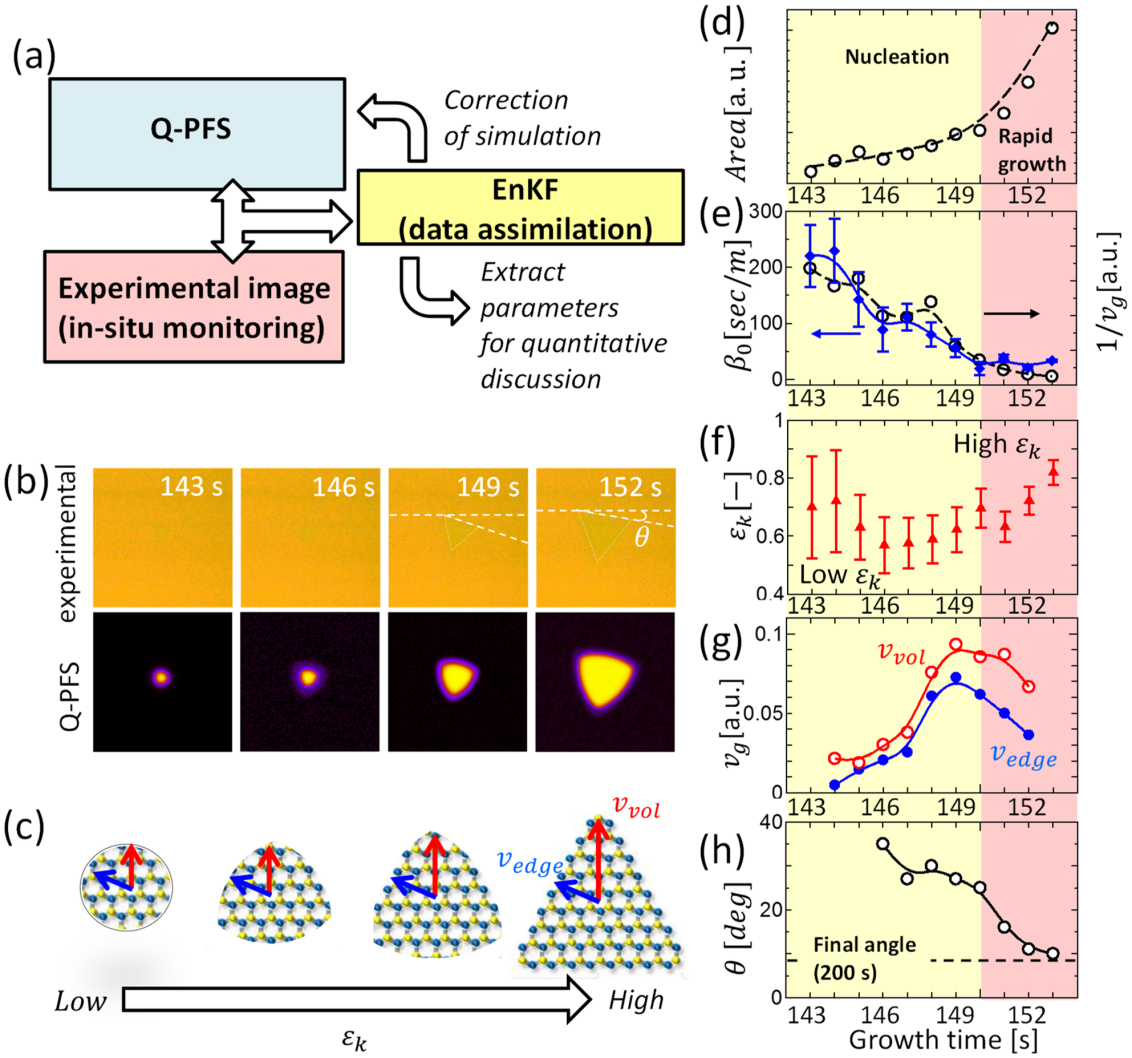
Data assimilation of Q-PFS and in-situ images. (**a**) Schematic of calculation flow. (**b**) Typical results of (up) experimental and (down) calculated images for monolayer WS_2_ growth. (**c**) Schematic of time evolution for the nucleation of WS_2_ under the different $$\varepsilon_{k}$$. (**d**–**h**) Plot of time evolution for (**d**) monolayer area (experiment), (**e**) $$1 / \beta_{0}$$ (calculation) and $$v_{g}$$ (experiment), (**f**) $$ \varepsilon_{k}$$ (calculation), (**g**) $$ v_{edge}$$ and $$v_{vol}$$ (calculation), (**h**) $$\theta $$(experiment).

The $\varepsilon_{k}$ value can be determined by the difference in growth speed between the edge ($v_{edg}$) and vortex ($v_{vort}$) of the triangle (Fig. 4c). At the initial stage, the difference in growth speed was not significant, resulting in a relatively low $\varepsilon_{k}$, whereas a significant difference in growth speed appeared after 149 s, increasing the $\varepsilon_{k}$ (Fig. 4g). This timing coincides well with that of the start of rapid growth ($\sim150 s$). These transitions can also be explained by the two-step nucleation. The relatively small difference in $\varepsilon_{k}$ at the initial stage may be due to the intermediate cluster covering the WS_2_ crystal in the two-step nucleation. Once the intermediate cluster is consumed, the direct supply of precursor droplets increases the growth speed dominantly for the vortex of the triangle, thus increasing $\varepsilon_{k}$. It should also be noted that in addition to the $\varepsilon_{k}$, crystal orientation ($\theta$) also drastically changes during the transition from nucleation to rapid growth (Fig. 4b). The $\theta$ changed about 20 deg from the nucleation to the rapid growth ($\sim150 s$) (Fig. 4h). After starting the rapid growth, the $\theta$ stabilized to the final angle (Fig. 4h). This may be explained by the surface tension balance changes due to the consumption of the intermediate clusters around this time ($\sim150 s$). This is very important finding for achieving twist angle-controlled growth of bilayer TMDs in the future. Finally, we should discuss about the possible structure of intermediate cluster. In the reaction discussed in this study, the precursor droplet can be thought as mixture of Na_2_WO_4_, Na_2_W_2_O_7_, and S_x_ by following the other group’s report^10,30^. Final product is WS_2_ crystal, whose density (WS_2_: 7.5 g/cm^3^) is much higher than that of precursors (Na_2_WO_4_: 4.179 g/cm^3^, Na_2_W_2_O_7_: 5.19 g/cm^3^, S: 1.957–2.07 g/cm^3^). Note that we use crystal density for precursors because liuid density is uncertain. Thus, it can be conjectured that the intermediate cluster may be the structure between Na_2_WO_4_ + Na_2_W_2_O_7_ + S_x_ and WS_2_, which may possess higher density than that of precursors. Further detailed investigations are needed to clarify the detailed structure of intermediate cluster, which can be the future work of this study.

In summary, we established an in-situ monitoring CVD and automated image analysis system to identify the growth dynamics of monolayer TMDs in detail. Direct visualization was realized for the nucleation of TMD, where a two-step nucleation, also known as non-classical nucleation, could be observed in the VLS growth of WS_2_. The detailed nucleation dynamics reveals that the temperature dependence of the incubation time of the WS_2_ intermediate clusters matches well with the TTT diagram, which corresponds to traditional bulk crystal growth. Data assimilation based on Bayesian inference was also carried out to extract quantitative measures of growth parameters from the in-situ monitoring images. Crystal anisotropy could be quantitatively obtained, which was observed to increase after the growth mode change from slow to rapid, supporting the existence of two-step nucleation in the VLS growth of WS_2_. We believe that our findings on non-classical nucleation in VLS WS_2_ growth can contribute significantly to improving the quality of TMDs, such as increasing single domain size, controlling the orientation of monolayer TMD, and realizing twist-angle controlled growth of bilayer TMD.

## Methods

### Chemical vapor deposition (CVD)

WS_2_ was synthesized by thermal CVD using WO_3_ as the tungsten source. Ar was used as the carrier gas at a flow rate of 150–500 sccm. Sulfur ($\sim$ 0.5 g) was placed in a Sulfur heater and WO_3_ ($\sim$ 40 mg) on a almina boat was set 2 cm downstream in the center of the CVD furnace. NaCl ($\sim$ 6 mg) was mixed with WO_3_ to enhance the evaporation. There is temperature gradient in our electric furnace. The substrate is placed near the end of electric furnace, which is about 3 cm away from the WO_3_. Thus, temperature of substrate is lower than that of WO_3_, which is referred as the temperature of main furnace.

### STEM measurements

The single crystalline structure of the triangular domain was confirmed by atomic-resolution Z-contrast STEM (FEI Titan G2 60-300 Cubed Double Corrector, 60 keV) (Fig. 1h,i).

### PL mapping

A J/Y PL system (HR800) was used for PL mapping. A semiconductor laser (532 nm) was used for the excitation.

### In-situ monitoring

The optical microscope was set above the main electrical furnace, which was a hand maid with a heater line (Fig. S5). Optical observation of the substrate surface during CVD growth was possible through the gap of the heater line. To independently control the substrate temperature from other conditions, such as the sulfur heater temperature, main furnace temperature, gas flow, and substrate position, a spot heater was placed outside the main electrical furnace. The substrate was placed in the downstream region of the main furnace.

### Automated image analysis

The image analysis of in-situ monitoring CVD was carried out using Open CV with a Python system (Fig. S2).

### Quantitative phase-field simulation

The quantitative phase-field model proposed by Bragard et al.^27^ was used for the simulation of WS_2_ growth under the modification of the description for the *N*-time symmetry. An order parameter *ϕ*, which takes a value of + 1 for the sold crystalline WS_2_ and − 1 for the liquid phase, was employed. *ϕ* changed from − 1 to + 1 continuously inside the interface. The time-evolution equation^29^ is given by

4 $$ \tau \left( {\mathbf{n}} \right)\frac{\partial }{\partial t} = \nabla \left[ {W\left( {\mathbf{n}} \right)^{2} \nabla \phi } \right] + \mathop \sum \limits_{i = x,y} \partial_{i} \left( {\left| {\nabla \phi } \right|^{2} W\left( {\mathbf{n}} \right)\frac{{\partial W\left( {\mathbf{n}} \right)}}{{\partial \left( {\partial_{i} \phi } \right)}}} \right) + \phi - \phi^{3} - \lambda \left( {1 - \phi^{2} } \right)^{2} u_{{{\text{int}}}} , $$

5 $$ W\left( {\mathbf{n}} \right) = W_{0} a_{c} \left( {\mathbf{n}} \right),\;\tau \left( {\mathbf{n}} \right) = \frac{1}{{a_{1}^{2} }}\frac{{W_{0}^{2} }}{{d_{0} }}\beta_{0} a_{c} \left( {\mathbf{n}} \right)a_{k} \left( {\mathbf{n}} \right), $$

6 $$ a_{c} \left( {\mathbf{n}} \right) = 1 + \varepsilon_{c} cos\left( {N\theta } \right),\;a_{k} \left( {\mathbf{n}} \right) = 1 - \varepsilon_{k} cos\left( {N\theta } \right). $$

Here **n** is the unit vector normal to the interface; *W*_0_ is the interfacial thickness; *λ* is the coupling constant given by *λ* = *a*_1_*W*_0_/*d*_0_, with $a_{1} = 5 \sqrt 2 / 8$, *d*_0_ is the capillary length defined as *d*_0_ = *σ*_0_ (*T*_*m*_*c*_*p*_/*L*^2^); *β*_0_ is the kinetic coefficient; *u*_int_ is the dimensionless undercooling at the interface; and *ε*_*c*_ and *ε*_*k*_ are anisotropy parameters of the interfacial energy and mobility, respectively. The thermodynamic and interfacial parameters are listed in Table 1. Equation (4) was discretized using a second-order finite-difference scheme with grid spacing *Δx* = 3.698 μm and solved using a first-order Euler scheme with a time step *Δ of t* = 1 × 10^−3^ s. We set *u*_int_ to be 3.1 × 10^−4^, assuming that the temperature of the interface is close to that of the substrate because it is not straightforward to specify the temperature of the interface during crystal growth.

**Table 1:** Input parameters for Q-PFS.

Parameter	Symbol	Value
Specific heat at constant pressure^31^	*Cp*	1.685 × 10^6^ (J/Km^3^)
Melting point	*T*_*m*_	1073 (K)
Latent heat	*L*	4.735 × 10^8^ (J/m^3^)
Interfacial energy	*σ*_0_	0.4412 (J/m^2^)
Anisotropy in interfacial energy	*ε*_*c*_	0.05

### Ensemble Kalman filter (EnKF)

Ensemble Kalman Filter (EnKF) is the Monte Carlo approximation of the Kalman filter^28^, which is widely employed for data assimilation in computational simulation and observation data. The EnKF conducts multiple simulations (called ensembles) with different parameters in parallel; this corresponds to the ensemble member of the probability distribution function of the state. The EnKF consists of cycles of prediction and filtering stages (Fig. S8). In the prediction stage, each simulation was performed independently and simultaneously. In the filtering stage, the parameters and probability distribution function of the state are updated according to the observation data using the Kalman gain filter^28,29^. The parameters are simultaneously estimated by repeating the prediction and filtering steps. The expected values of the parameters are obtained from the ensemble average at each filtering step as sequential data with respect to time. Here, 100 ensemble Q-PFS are performed in parallel, and in-situ monitoring images at every 1 s are used for the filtering step. In-situ monitoring images are converted in advance to the order parameter *ϕ* ranging from − 1 to + 1 in the 100 × 100 mesh space to perform the filtering step. The order parameter at all mesh points as well as two parameters, kinetic coefficient *β*_0_ and kinetic anisotropy *ε*_*k*_, are employed as state variables. Therefore, these two parameters and the morphology of the WS_2_ crystals were estimated after data assimilation.

## Supplementary Information

Supplementary Information 1.

Supplementary Video 1.

Supplementary Video 2.
