# Supplementary material for: Non-classical nucleation in vapor–liquid–solid growth of monolayer WS2 revealed by in-situ monitoring chemical vapor deposition
Source: Sci Rep. 2021 Nov 15;11:22285. doi: 10.1038/s41598-021-01666-9 (PMC8593054; doi:10.1038/s41598-021-01666-9)
Supplement: Supplementary file 1 — Supplementary Information 1. [file 41598_2021_1666_MOESM1_ESM.docx]

**Supporting Information**

**Non-classical nucleation in vapor-liquid-solid growth of monolayer WS_2_ revealed by in-situ monitoring chemical vapor deposition**

Xiaoming Qiang^1^, Yuta Iwamoto^1^, Aoi Watanabe^2^, Tomoya Kameyama^1^, Xing He^1^, Toshiro Kaneko^1^, Yasushi Shibuta^2^, and Toshiaki Kato^1,^*

^1^Graduate School of Engineering, Tohoku University, 980-8579 Sendai, Japan

^2^Department of Materials Engineering, The University of Tokyo, 7-3-1, Hongo, Bunkyo-ku, Tokyo 113-8656, Japan

*Corresponding Author e-mail: kato12@ecei.tohoku.ac.jp

Fig. S1. Schematic of in situ monitoring CVD system and plot of substrate temperature as a function of spot heater current.

Fig. S2. Typical results of auto image analysis. (a) Original microscope image of TMD obtained by in situ monitoring. (b–g) Analyzed results of (b–d) monolayer and (e–g) multilayer process after (b,e) threshold filtering, (c,f) binarization, and (d,g) extraction of edges.

Fig. S3. Typical plot of (a) area ($A$) vs. time ($t$) and (b) $dA / dt$ vs. $t$ obtained by automated image analysis.

Fig. S4. Comparison of pixel size and particle size obtained by automated image analysis.

Fig. S5. Typical in situ monitoring image of TMD growth under oversupply of precursors (a: 138 s, b: 148 s, c: 158 s, d: 178 s, e: 198 s, f: 218 s). Arrows show the position of monolayer WS_2_.

Fig. S6. Typical scanning electron microscopy (SEM) image of monolayer WS_2_ grown without salt assist.

Fig. S7. Plot of *T*_sub_ as a function of ln(*t*_2_). Dashed line is drawn to guide the eye.

Fig. S8.　Schematic of data assimilation of Q-PFS and in situ images.

Fig. S9. Estimation of (a) kinetic coefficient *β*_0_ and (b) anisotropy parameter of mobility *ε_k_* from the data assimilation with various initial guess configurations.

**Supplementary movies**

Movie 1. Original movie of WS_2_ growth. Time evolution of optical microscope images of substrate during WS_2_ growth were continuously measured by in-situ monitoring CVD.

Movie 2. Analyzed movie of WS_2_ growth. Original movie and edge of precursors/WS2 were overlapped, which was obtained by auto-image analysis.
